# Potential biomarkers for southern African hunter-gatherer arrow poisons applied to ethno-historical and archaeological samples

**DOI:** 10.1038/s41598-023-38735-0

**Published:** 2023-07-23

**Authors:** Sven Isaksson, Anders Högberg, Marlize Lombard, Justin Bradfield

**Affiliations:** 1 The Archaeological Research Laboratory, Department of Archaeology and Classical Studies, Stockholm University, Stockholm, Sweden; 2 Department of Cultural Sciences, Faculty of Arts and Humanities, Linnaeus University, Kalmar, Sweden; 3 Palaeo-Research Institute, University of Johannesburg, Johannesburg, South Africa

**Keywords:** Biochemistry, Environmental social sciences, Biomarkers

## Abstract

The detection of complex poison recipes applied to ancient hunting weapons has the potential to provide important insights into traditional pharmacological knowledge systems. Yet, recipes comprising many ingredients can be challenging to decipher, especially in older samples that have undergone biodegradation. We present the results of our attempt to analyze samples of poison collected from nineteenth and twentieth century arrowheads from southern Africa, and from a 1000-year-old archaeological bone point. The arrow poison residues and reference samples were analyzed by Attenuated Total Reflectance Fourier Transform Infrared Spectroscopy (ATR FTIR) and Gas Chromatography Mass Spectrometry (GC–MS). The ATR FTIR analysis is primarily able to separate between different arrow poison binder recipes. The extractives identified by GC–MS analysis consist of a multitude of components from both binders and active substances, confirming and adding to the results from the ATR FTIR analyses. We discuss the results in terms of potential biomarkers for arrow poisons in organic residue analyses of archaeological artefacts; that residues of toxic cardiotonic glycosides can be detected on curated and excavated arrow tips of between about 1000 and 100 years old, serves as proof of concept for working with older materials in the future.

## Introduction

One of the enduring fascinations with the technologies of hunter-gatherer societies is their poisoned weapons^1–3^. The San of southern Africa are renowned for their use of poisoned arrows to hunt a wide range of animals, which they would often track for days while the poison took effect^4^. Indeed, the lightweight, flimsy arrows of the San would likely be ineffective on larger animals without the application of poison^5,6^. Precisely when the Stone Age hunter-gatherer ancestors of the San started using poison as an aid to hunting is a matter of considerable interest and debate.

Based on their tip cross-sectional areas, Lombard^7^ speculated that poisoned bone arrow tips could have been used before 70 ka in southern Africa. One of these points has been found in ~ 61 ka deposits at Klasies River Main site, Eastern Cape Province, South Africa^8^. It is coated in a black residue, rich in organic components. The placement of this residue coating is suggestive of poison application, but the precise chemical composition of the residue has not yet been established. At Border Cave, in KwaZulu-Natal, South Africa, toxic plant-based compounds have been identified on a wooden applicator stick dating to 24 ka^9^. Some of these toxic compounds, which include ricinoleic acid, are thought to be the oxidative by-products of the toxin ricin, found in castor beans. It is possible, however, that these by-products could have come from a similar, but unrelated, plant species; the *Abrus precatorius* plant, which grows naturally in the area and is just as toxic, if not more so^10–12^. Bone points, covered in what is thought to be poison, have been recovered from 13 ka levels at Kuumbi Cave in Zanzibar, but no chemical confirmation of these residues was undertaken^13^.

The challenge with accurately identifying the chemical signatures of organic compounds preserved as archaeological residues is precisely because they degrade into constituent parts through time. Coupled to this problem is the fact that most arrow poisons, at least those we know of from the ethno-historical record, were actually complex recipes, comprising many ingredients and preparatory steps^10,14,15^, and which differed from region to region^16,17^. Some non-toxic ingredients were also added for their adhesive properties, or simply because they were believed to impart certain effects^18–20^. For example, trapdoor spiders were ground up whole and mixed with other ingredients^21^. This added nothing to the toxicity of the mixture^22,23^, but would introduce many hundreds of proteins and polypeptides into the mix. Once these mixtures biodegrade it becomes very difficult to reconstruct the parent compounds, particularly when several such compounds may be present.

Current biochemical knowledge of African arrow poisons comes from studies that have sought to characterize the parent compounds from fresh ingredients^11,14,22–24^. These studies necessarily focused on ethno-historically recorded hunting poisons, which, in the case of southern Africa, are confined to the dry, western regions of the sub-continent. There are many toxic plants (and animals, e.g., the red-banded rubber frog) in the eastern districts which are suitable for hunting poisons, but for which we have no recorded evidence of use^10^. In a study by Shaw and colleagues poison on 80-year-old arrows was found to be still pharmacologically active^25^. The longevity of these poisons was thought to be due to plant-based ingredients, rather than the diamphotoxin from the *Diamphidia* grub, which was the main ingredient. Despite several recent chemical studies on organic poisons and adhesive residues, predominantly of plant origin^9,18,26,27^, much work remains to be done to recognise the biodegradative pathways of organic compounds and the effects of preparation procedures on such pathways.

**Figure 1:**
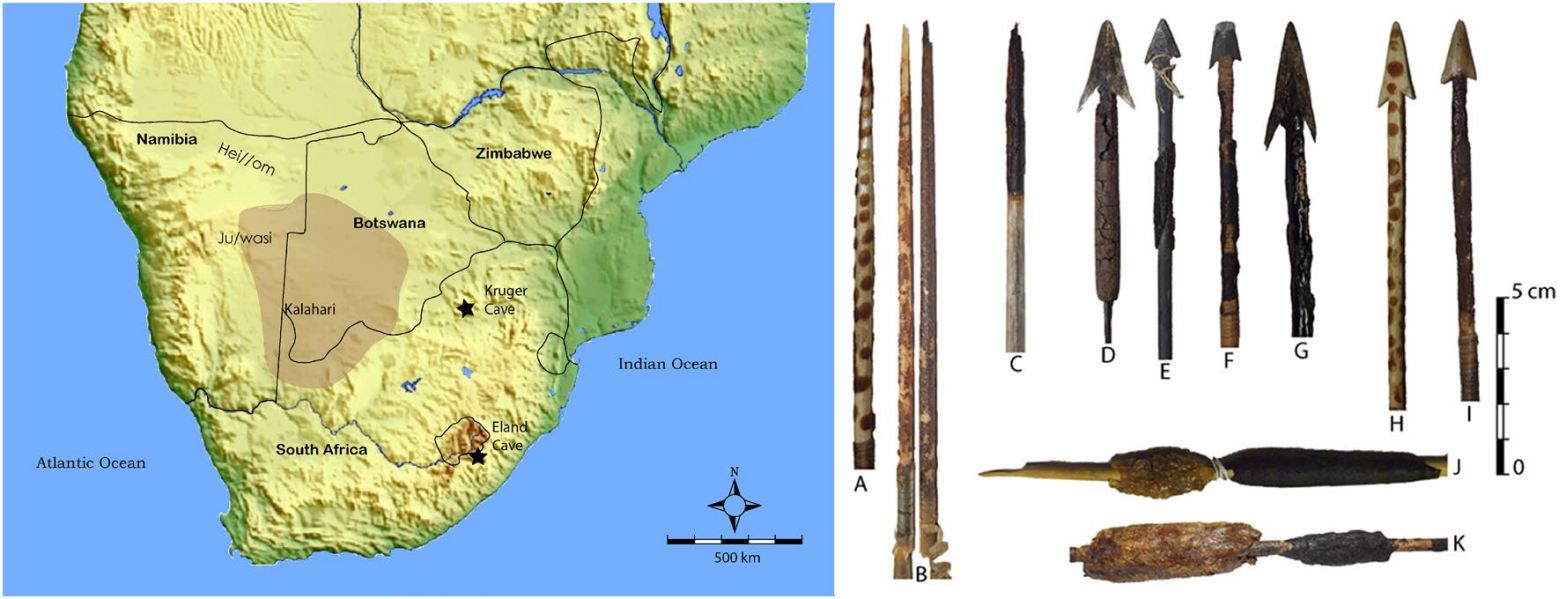
Map of southern Africa showing the regions from which the arrowheads were collected. On the right, a selection of arrows sampled for poison analysis. Simple bone lanceolate points: (**A**) ET 3413; (**B**) MM 1-67-600. Bone point with metal-tipped inset: (**C**) SMP 2528. Tanged metal arrowheads: (**D**) MA 1948-61; (**E**) ET 6577; (**F**) ET 5099/3; (**G**) MA 1948-6L. Tanged bone arrowheads: (**H**) ET 5109/4; (**I**) ET 5113. Poison and adhesive storage sticks: (**J**) ET 1989/15/9; (**K**) MM 40-69-2805.

To this end we present a biochemical study of poison residues from nineteenth and twentieth century arrowheads collected from northern Namibia and the Kalahari (Fig. 1). It is useful to know whether people were using complex poison recipes as a hunting aid, and ultimately to assess the time depth of such innovation, because it speaks directly to the development of complex cognition in *Homo sapiens*^28,29^. We include a single archaeological specimen from Kruger Cave in the Magaliesberg region of South Africa. This poisoned arrowhead is approximately 1000 years old. We hope that by analyzing increasingly older poison residues we will eventually gain a better understanding of how these complex organic mixtures decompose in order to be able to identify potential biomarkers and chemical fingerprints that can be used to identify arrow poisons on older archaeological specimens. To do so we first perform a general characterization of the arrow poison residues using Fourier Transform Infrared spectroscopy (FTIR)^30,31^. IR-spectra of samples were compared by an arithmetic data-point by data-point comparison resulting in a correlation coefficient (Pearson r) matrix that was investigated for group structure using a hierarchical cluster analysis^31^. The Pearson r was chosen since the algorithm used (a linear regression) accounts for factors such as baseline drift and difference in scaling between samples, making baseline corrections or normalization unnecessary. Also, since the calculated match values are correlation coefficients they are absolute values with statistical significance, instead of some measure of relative best fit. However, this method only provides an indication of group structure rather than a rigid model of the data^32^. To acquire such a model the spectral data of samples and reference materials were subjected to PCA (Principal Component Analysis) and DFA (Discriminant Function Analysis). The purpose of the PCA was to reduce the number of variables (i.e. wavenumbers) and to detect structure in the relationships in absorbance at different wavenumbers (i.e. chemical bonds and functional groups). The relevant principal components of the reference materials were then used as variables in a DFA to produce a classification model used to classify the unknown samples. We then proceed by applying the standard procedure of organic residue analysis for the investigation of lipid residues; ultrasonic-assisted solvent extraction and Gas Chromatography Mass Spectrometry (GC–MS)^33^. We chose this procedure since lipids, and compounds biochemically and functionally related to lipids, persist in archaeological materials over long periods of time. Since the composition of arrow poison is unknown, we adopt an explorative non-targeted approach, investigating to what extent extractives from arrow poison residues can be identified using this procedure.

## Results

### Statistical analysis of IR-spectra

**Figure 2:**
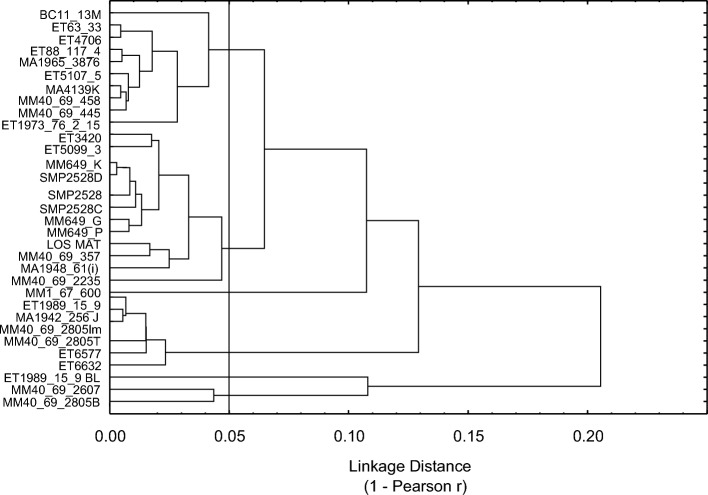
Tree diagram based on a correlation coefficient matrix using a linear regression algorithm of IR spectra of the samples.

Since Pearson r is a measure of similarity and the cluster analysis needs a measure of dissimilarity it was converted to (1 − r) and used as the linkage distance in the cluster analysis. It is unlikely that dissimilar samples will display correlation coefficients higher than 0.95. Consequently, a linkage distance of 1 − 0.95 = 0.05 was chosen as the cut-off distance for meaningful group structure; this result in four groups of ten, thirteen, six and two samples respectively, and two single samples (MM 1-67-600 and ET1989/15/9 black) that don’t group with any of the other samples (Fig. 2). The result clearly shows that there is group structure in the data.

The PCA of the IR-spectra of reference materials and samples showed that the first four principal components had high loadings (> + 0.7: < − 0.7) at specific regions of the spectra. The first principal component explains 47.2% of the total variation in the data set and primarily accounts for variation in the 1793–2487 and the 3645–3992 cm^−1^ regions of the spectra, which corresponds to background variations between samples. The second principal component explains 21.4% of the total variation in the data set with a strong positive loading in the 1176–1215 and 1716 cm^−1^ region of the spectra and with a strong negative loading in the 3066–3529 cm^−1^ region. IR absorption in the 1176–1215 cm^−1^ region can have a number of different sources but most distinctive are C–C stretching, C–O stretching and OH deformation adsorption. Adsorption around 1716 cm^−1^ is characteristic of C=O stretching, and the broad adsorption in the 3066–3529 cm^−1^ region is characteristic of OH and NH stretching absorption. The third principal component explains 12.3% of the total variation with a strong positive loading in the 1485–1523 cm^−1^ region, characteristic for O–H bending and C–H deformation adsorption, and a strong negative loading in the 868-984 cm^−1^ region, characteristic for adsorption associated with CO_3_^2−^ and NO_3_^−^. The fourth principal component explains 6.3% of the total adsorption with a strong positive adsorption in the 2565–2873 and 2950–2989 cm^−1^ regions, characteristic for C–H and O–H stretching adsorptions, and a strong negative adsorption in the 598–752 cm^−1^ region, characteristic of C–S and N–O stretching adsorption as well as adsorption associated with SO_4_^2−^.

Since the first principal component was associated with background variation this factor was excluded from the DFA. In the model produced by the DFA, the first two roots cover 99.5% of the variation in the three principal components (Table 1). The first root has a strong positive loading from PC 2 and 4, and a moderate negative loading from PC 3, while the second root has strong negative loadings from PC 2 and 3.

**Table 1:** Loadings of each principal component to each of the three roots in the discriminant factor analysis.

	Discriminant factor loadings		
	Root 1	Root 2	Root 3
PC 2	1.23	− 0.96	− 0.29
PC 3	− 0.67	− 1.10	0.24
PC 4	1.65	0.02	0.58
Eigenvalue	9.34	5.25	0.07
Cum.Prop.	0.64	0.995	1.00

*Cum. Prop.* cumulative proportion.

**Figure 3:**
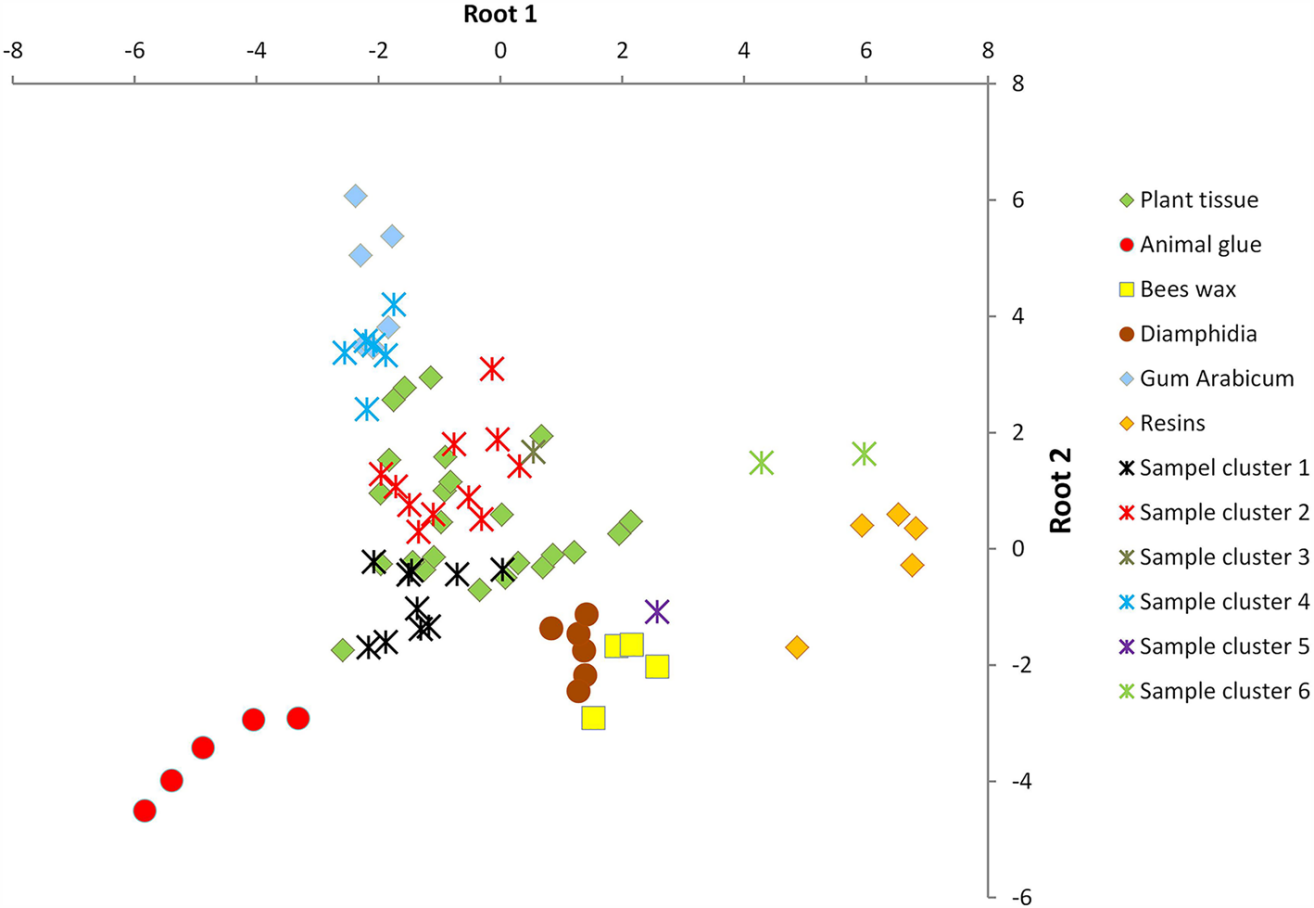
Scatterplot of the two roots from the discriminant factor analysis of the reference materials and the samples. Sample groupings refer to groupings in this figure: Sample cluster 1 = BC11-13M, ET63/33(f), ET 88/117/4, ET1973/76/2, ET4706, ET5107/5, MA1965-3876, MA4139(K), MM40-69-445, MM40-69-458. Sample cluster 2 = ET3420, ET5099/3, MA1948-61(I), MM40-69-357, MM40-69-2235, MM649_G, MM649_K, MM649_P, SMP2528a, SMP2528c, SMP2528d, SMP loose. Sample cluster 3 = MM1-67-600. Sample cluster 4 = ET1989/15/9 (light), ET6577, ET6632, MA1942-256 (J), MM40-69-2805 (light), MM40-69-2805 (transparent). Sample cluster 5 = ET1989/15/9 (dark). Sample cluster 6 = MM40-69-2607, MM40-69-2805 (black).

Consulting the spectral component loadings from the PCA this suggests that spectra with pronounced adsorption from C–H, C–C and C–O stretching should have positive scores in Root 1 while spectra with pronounced adsorption from N–O and N–H stretching adsorption should have a negative score in Root 1. Spectra with a pronounced adsorption from O–H stretching should have a positive score in Root 2 and spectra with pronounced adsorption from C=O and C–C stretch should get a negative score in Root 2. Testing the model by comparing observed versus correct classification show that the model is 93.2% correct, and as can be seen in Fig. 3 it separates the reference materials in the plot. The protein rich animal glues have negative scores on Root 1 (N–O and N–H bonds) and the increasing occurrence of C–O, C–H and C–C bonds in carbohydrates, lipids and resins respectively separate the plant tissues, fat- and wax-rich tissues and pitches with high scores along the same axis. The OH-rich polysaccharides in natural gum have high scores on Root 2, separating them from other carbohydrates and more carbon-chain rich materials such as fats and resins. The fat-rich *Diamphidia* and the bees wax reference materials scatter very close.

Adding the samples to the scatterplot, marked as per group in the hierarchical cluster analysis (sample clusters, Fig. 2) shows that most of the samples plot together with the reference materials of plant tissue (Fig. 3). This is further emphasized when using the DFA model to calculate a posteriori probabilities for the samples in relation to the different groups of reference materials (Table 2). Note that the reference material constitutes the whole “reality” for the model and it is quite unlikely that the model would give the result “none of these groups” for a sample of a material not included in the model unless this material is chemically very different from all the reference materials. Also, the reference materials are singular substances whereas the samples most likely are mixtures that may cause scatters between well separated groups of reference materials. None of the samples are classified as hide glues, which comes as no surprise since there is, to our knowledge, no reference to hide glue in the southern African context. One sample (ET1989/15/9) is classified as between bees wax and *Diamphidia*. Four samples are classified as predominantly gum, while another two comprise predominantly resins. Sample clusters 1–6 are well separated in the scatterplot (Fig. 3). Sample cluster 1 has lower scores on Root 2 and most of the plant references they scatter close to are *Euphorbia*. Sample cluster 2 and 3 scatter slightly higher on Root 2 and most of the plant reference they scatter close to are *Adenium*. Sample cluster 4 is even higher on Root 2 and is close to the *Gum Arabicum* references. The two specimens in sample cluster 6 both have higher scores in Root 1 and are closest to the resin references (cf. Table 2) but still far from these in Fig. 3 suggesting that they consists of a type of resin other than those in the reference material, or that they are mixed with something more carbohydrate-rich. It appears that the specimens in sample cluster 4 have plant gum as a main ingredient though with a varying degree of mixture with more cellulose rich materials; the specimens in sample clusters 2 and 3 have more cellulose rich main components (e.g., *Sansevieria*, in addition to *Adenium*); and the specimens in sample cluster 1 have more waxy plants (such as *Euphorbia*) as main ingredients.

**Table 2:** Classification of cases based on the discriminant factor analysis.

Artefact	Animal glue	Bees wax	Diamphidia	Gum arabic	Resins	Plant
BC11-13M (sample cluster 1)	0.074	0.001	0.030	0.000	0.000	**0.896**
ET63/33(f) (sample cluster 1)	0.000	0.000	0.001	0.000	0.000	**0.999**
ET88/117/4 (sample cluster 1)	0.000	0.000	0.005	0.000	0.000	**0.995**
ET1973/76/2 (sample cluster 1)	0.000	0.007	0.069	0.000	0.000	**0.925**
ET1989/15/9 (dark) (sample cluster 5)	0.000	*0.446*	**0.537**	0.000	0.001	0.016
ET1989/15/9 (light) (sample cluster 4)	0.000	0.000	0.000	**0.967**	0.000	0.033
ET3420 (sample cluster 2)	0.000	0.000	0.004	0.000	0.000	**0.995**
ET4706 (sample cluster 1)	0.000	0.000	0.006	0.000	0.000	**0.994**
ET5099/3 (sample cluster 2)	0.000	0.000	0.001	0.000	0.000	**0.998**
ET5107/5 (sample cluster 1)	0.014	0.001	0.037	0.000	0.000	**0.948**
ET6577 (sample cluster 4)	0.000	0.000	0.000	**0.891**	0.000	*0.109*
ET6632 (sample cluster 4)	0.000	0.000	0.000	*0.223*	0.000	**0.777**
MA1942-256 (J) (sample cluster 4)	0.000	0.000	0.000	**0.958**	0.000	0.042
MA1948-61(I) (sample cluster 2)	0.000	0.000	0.000	*0.224*	0.000	**0.776**
MA1965-3876 (sample cluster 1)	0.000	0.001	0.022	0.000	0.000	**0.977**
MA4139 (K) (sample cluster 1)	0.001	0.003	0.067	0.000	0.000	**0.929**
MM1-67-600 (sample cluster 3)	0.000	0.000	0.001	0.000	0.000	**0.999**
MM40-69-357 (sample cluster 2)	0.000	0.000	0.000	0.003	0.000	**0.997**
MM40-69-445 (sample cluster 1)	0.000	0.001	0.028	0.000	0.000	**0.971**
MM40-69-458 (sample cluster 1)	0.000	0.004	0.074	0.000	0.000	**0.922**
MM40-69-2235 (sample cluster 2)	0.000	0.000	0.001	0.002	0.000	**0.997**
MM40-69-2607 (sample cluster 6)	0.000	0.002	0.001	0.000	**0.996**	0.001
MM40-69-2805 (black) (sample cluster 6)	0.000	0.000	0.000	0.000	**1.000**	0.000
MM40-69-2805 (light) (sample cluster 4)	0.000	0.000	0.000	**0.973**	0.000	0.027
MM40-69-2805 (transparent) (sample cluster 4)	0.000	0.000	0.000	**0.996**	0.000	0.004
MM649 G (sample cluster 2)	0.000	0.000	0.000	0.002	0.000	**0.998**
MM649 K (sample cluster 2)	0.000	0.000	0.000	0.000	0.000	**0.999**
MM649 P (sample cluster 2)	0.000	0.000	0.000	0.000	0.000	**1.000**
SMP2528a (sample cluster 2)	0.000	0.000	0.000	0.001	0.000	**0.999**
SMP2528c (sample cluster 2)	0.000	0.000	0.000	0.000	0.000	**0.999**
SMP2528d (sample cluster 2)	0.000	0.000	0.001	0.000	0.000	**0.999**
SMP loose (sample cluster 2)	0.000	0.000	0.000	0.004	0.000	**0.996**

In the columns are the posteriori probabilities, the probability for a sample to belong to that class of reference materials, based on the DFA model of the PCs of the IR-spectra. The highest a posteriori probability for each sample is marked with bold, if there is another probability higher than 0.1 this has been marked in italics. Sample cluster number refers to the clusters in Fig. 3.

### Gas chromatography-mass spectrometry

The gas chromatography mass spectrometer analysis (GC–MS) of extracts from these samples produced a fairly complex set of data. In the 31 samples that produced results from this analysis ~ 240 different compounds were detected, most of them as trimethylsilyl esters or ethers. Using the Masshunter and NIST Mass Spectral Search Program software it was possible to identify most of these compounds to class and many to specific components, although it was not possible to identify all of the specimens (Table 3). Two samples stand out in the number of unidentified components: ET1989/15/9 and MM40-69-2805 (transparent). MM40-69-2805 (transparent) is a piece of transparent material on that sample and also the sample with the highest amount of identified contaminants and can be excluded as a piece of lacquer or glue. Sample ET1989/15/9 plotted close to beeswax and *Diamphidia* in the FTIR analysis (Fig. 3) but, after the unidentified compounds, is dominated by a range of pentacyclic triterpenoids in the GC–MS analysis (Table 3).

**Table 3:** Summary of compound classes identified by GC–MS in the samples.

	Di- and triols	Short-chain organic acids	Lipids (fatty acids and related compounds)	Carbohydrates	Terpenoids	Steroids (except sterols)	Unidentified	Contaminants
BC11-13M (sample cluster 1)	0	0	60.5	0	15.3	17.4	6.8	0
ET1973/76/2 (sample cluster 1)	0	0	71.0	26.9	0	0	2.1	0
ET1989/15/9 (sample cluster 5)	0	0	3.3	0	28.3	19.8	48.3	0
ET3420 (sample cluster 2)	0	0	29.8	69.2	0	0	0.3	0
ET4706 (sample cluster 1)	1.5	2.5	33.5	59.7	0	0	2.7	0
ET5099/3 (sample cluster 2)	1.9	7.9	58.5	27.9	0	0	3.8	0
ET5107/5 (sample cluster 1)	0.4	0	63.4	33.8	0	1.4	1.0	0
ET63/33(f) (sample cluster 1)	0	0. 6	60.6	27.3	0	0	11.6	0
ET6577 (sample cluster 4)	0	0	94.2	2.0	0.5	0	2.6	0.6
ET6632 (sample cluster 4)	4.7	2.5	69.6	19.2	0	0	1.2	2.8
ET88/117/4 (sample cluster 1)	0	0	22.3	77.7	0	0	0	0
MA1942-256(J) (sample cluster 4)	0	4.2	47.5	35.8	2.8	0.7	6.3	2. 8
MA1948-61(I) (sample cluster 2)	1.0	0	37.0	58.5	0	0	2.0	1.5
MA1965-3876 (sample cluster 1)	0	0	59.4	40.4	0	0	0	0.2
MA4139 (K) (sample cluster 1)	0	0	66.5	32.1	0	0	1.4	0
MM1-67-600 (sample cluster 3)	4.7	0	52.0	14.2	0	3.1	19.9	6.2
MM40-69-2235 (sample cluster 2)	8.0	1.3	45.5	38.4	0	1.2	1.3	4.4
MM40-69-2607 (sample cluster 6)	0.8	0	29.4	1.7	0	56.5	11.6	0
MM40-69-2805 (black) (sample cluster 6)	1.4	0	78.6	8.3	0	4.0	7.6	0
MM40-69-2805 (light) (sample cluster 4)	0.4	0.6	44.7	39.9	2.0	0	5. 9	6.5
MM40-69-2805 (transparent) (sample cluster 4)	7.1	3.1	12.8	8.7	2.0	3.0	37.8	25.5
MM40-69-357 (sample cluster 2)	2.3	0	11.2	14.4	3.9	52.3	13.8	0
MM40-69-445 (sample cluster 1)	1.2	0.4	43.1	54.1	0	0	1.3	0
MM40-69-458 (sample cluster 1)	0	0	73.0	27.0	0	0	0	0
MM649 G (sample cluster 2)	2.0	6.0	79.4	3. 5	0	0.6	4.2	4.4
MM649 K (sample cluster 2)	1.8	5.1	87.8	1.2	0	1.1	1.4	1.6
MM649 P (sample cluster 2)	0.5	1.0	95.3	0.8	0	0	0. 8	1.7
SMP2528a (sample cluster 2)	0	1.7	1.6	4.6	83.4	5.2	2.3	1.2
SMP2528c (sample cluster 2)	0	11.1	52.0	17.7	4.6	3.2	3.4	8.0
SMP2528d (sample cluster 2)	0	27.7	24.2	29.8	0	0	15.7	2.5
SMP loose (sample cluster 2)	1.0	2.5	6.7	4.9	56.2	21.8	5.9	1.1

The numbers given are the area percentage of the Total Ion-Chromatograms for each sample. Sample cluster number refers to the clusters in Fig. 3.

**Figure 4:**
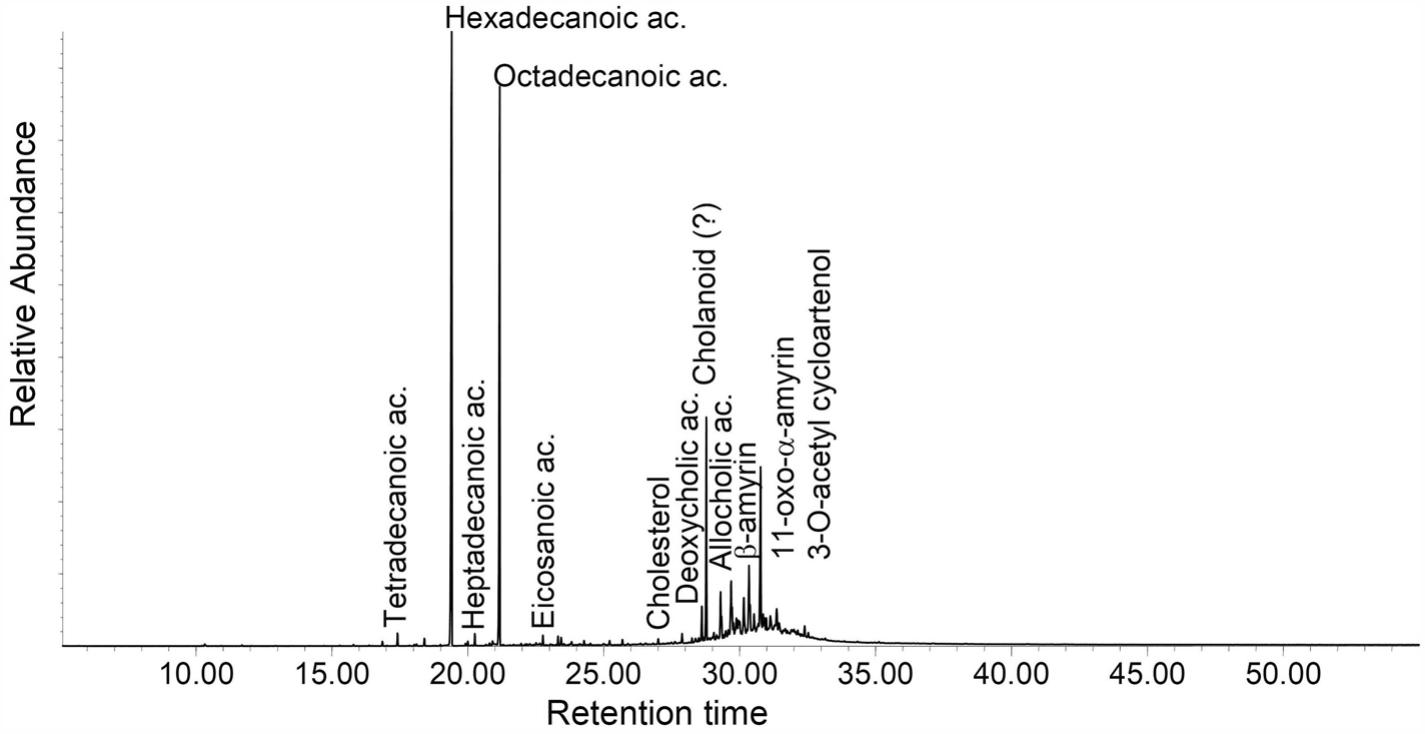
Total ion chromatogram of sample BC11-13M (Date: 1020 ± 70 BP). The sample is dominated by fatty acids and also contains steroidal and terpenoid components.

It is obvious that the 1000-year-old sample from Kruger Cave in the Magaliesberg (BC11-13M, Fig. 4) deviates from the other samples in that it contains none of the more water-soluble compound classes as seen in Table 3 (di- and triols, short-chain organic acids and carbohydrates). The samples classified as plant tissue and gums from the FTIR analysis (Sample cluster 1, 2 and 4 in Fig. 2) are also the samples containing the highest amounts of carbohydrates, and the samples classified as having more waxy plants (Sample cluster 1 in Fig. 2) are also the ones with generally higher yields of fatty acids and related compounds. Carbohydrates are an abundant class of organic materials in nature but carbohydrates have relatively little origin-dependent variability and are therefore a relatively poor source for information in archaeological materials^34^. The carbohydrates detected are a variety of C3-C6 monosaccharides and polyols, glyceryl glycosides and disaccharides.

**Figure 5:**
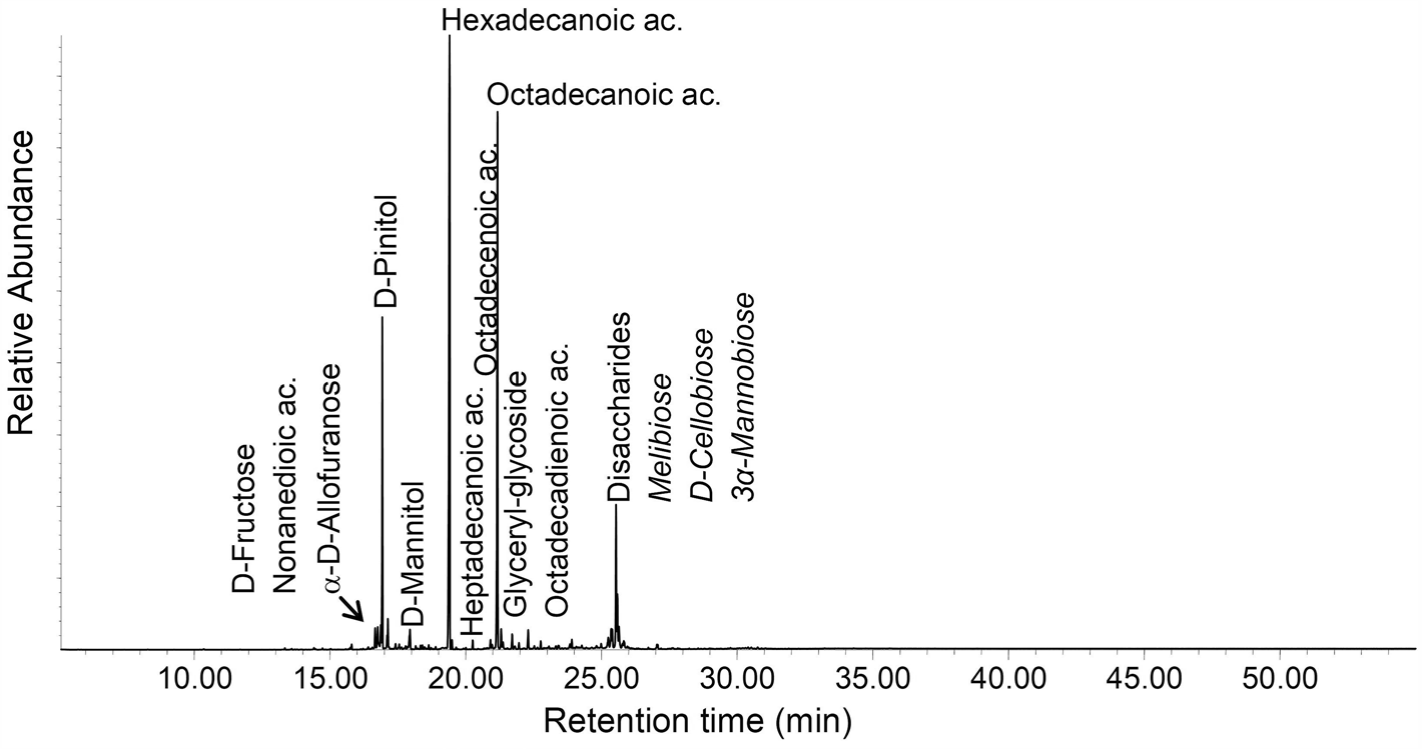
Total ion chromatogram of sample MA 4139K (Date: Pre 1936). This is an example of a sample dominated by lipids, probably of primarily plant oil origin, and mono- and disaccharides.

Lipid residues, on the other hand, are better studied^35^. The lipids are dominated by free fatty acids of chain-lengths ranging from C9 to C32 but dominated by Palmitic (C16) and Stearic (C18) acid. The presence of decomposition intermediates such as mono- and diacylglycides, β-hydroxy fatty acids and mid-chain dihydroxy fatty acids, show that the lipids are decomposing. Most specimen (n = 21) have a fatty acid distribution suggestive of a plant fat or oil origin, being clearly dominated by Palmitic acid as illustrated by a high Palmitic-to-Stearic (P/S > 1.3) acid ratio (Table 4). P/S is the ratio of palmitic to stearic acid, commonly higher than 1.3 in residues from plant oils but could also indicate aquatic animal fats. Though the ratio given here (P/S > 1.3) is valid for lipid residue ratios of fatty acids, which are in general susceptible to decompositional processes, individually they are only indicative of origin and need to be evaluated in context of other components. Fifteen specimens also contain short-chain dicarboxylic acids (C8-10), substances formed from drying oils, and in particular the presence of Azelaic acid (nonanedioic acid, C9) is indicative of the presence of a drying oil^36–38^. Azelaic acid is a common decomposition product from unsaturated fatty acid, in particular plant oils. Traces of the monounsaturated Oleic acid (n = 22) and diunsaturated Linoleic acid (n = 6) were also detected (cf. Fig. 5). Linoleic acid is a diunsaturated fatty acid common in several plant oils. Phytosterols are sterols produced by plants. Plant wax residues are primarily found as distributions of long-chain fatty acids, long-chain alkanols and a number of pentacyclic triterpenoids. Also present is D-Pinitol, which is a cyclitol common in plants of the *Leguminosae* and *Pinaceae* families^39^.

**Table 4:** Summary of lipids identified in the extracts from the arrow poison samples and P/S value rations.

	P/S > 1.3 Plant	Azelaic acid	Linoleic acid	Phytosterols	Plant wax	P/S < 1.3 Animal	Cholesterol
BC11-13M	X	–	–	–	–	–	X
ET1973/76/2	X	X	X	–	–	–	–
ET1989/15/9	–	–	–	–	–	–	–
ET3420	X	X	X	–	–	–	–
ET4706	–	X	X	–	–	X	–
ET5099/33	X	X	X	–	–	–	–
ET5107/5	–	–	–	–	–	X	X
ET63/33(f)	X	X	–	–	X	–	–
ET6577	X	X	–	X	X	–	–
ET6632	X	X	–	–	–	–	–
ET88/117/4	X	–	–	–	–	–	–
MA1942-256(J)	X	–	–	X	–	–	–
MA1948-61(I)	X	–	–	–	–	–	–
MA1965-3876	X	–	–	–	–	–	–
MA4139 (K)	X	X	X	–	–	–	–
MM1-67-600	X	X	X	X	–	–	X
MM40-69-2235	X	X	–	–	–	–	–
MM40-69-2607	–	–	–	–	X	–	–
MM40-69-2805 (black)	–	X	–	–	X	X	–
MM40-69-2805 (light)	X	–	–	–	–	–	–
MM40-69-2805 (transparent)	–	–	–	–	–	–	–
MM40-69-357	X	X	–	–	–	–	–
MM40-69-445	X	X	–	–	–	–	–
MM40-69-458	X	–	–	–	–	–	–
MM649 g	–	X	–	–	–	X	X
MM649 k	–	–	–	–	–	X	–
MM649 p	–	–	–	–	–	X	–
SMP2528a	X	–	–	–	–	–	–
SMP2528c	X	–	–	–	–	–	–
SMP2528d	–	X	–	–	–	X	–
SMP loose	X	–	–	–	–	–	–

Phytosterols were detected in four samples. In three specimens, distributions of long-chain (C > 20) fatty acids and alkanols were detected; traces of plant cuticle waxes^40,41^. Only a few specimens (n = 7) show fatty acid distributions suggestive of an animal origin, having a higher contribution of Stearic acid in relation to Palmitic acid. Cholesterol has been identified in five samples. This is a predominantly animal sterol but is also a major component in human skin lipids and can thus be trace of handling^42^.

**Figure 6:**
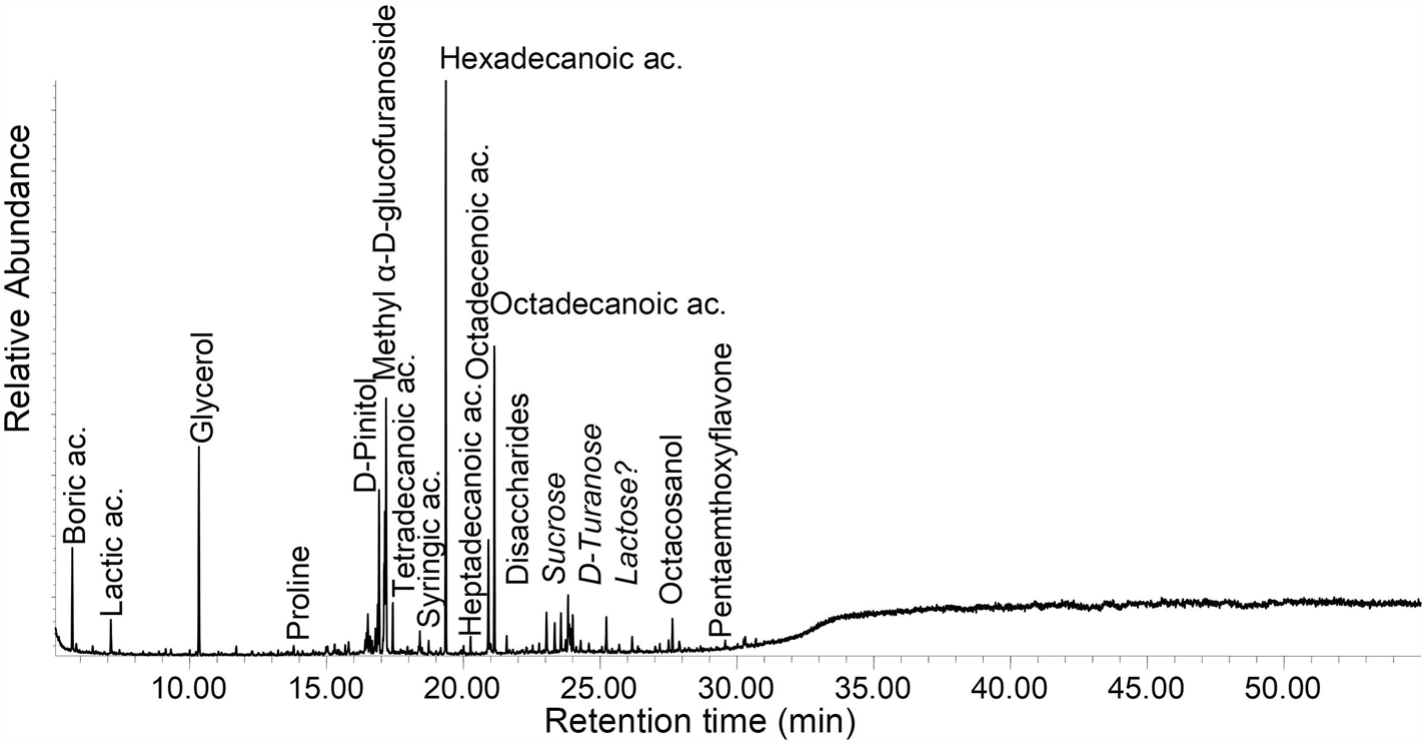
Total ion chromatogram of sample MM 40-69-2235 (Date: 1920s). This is an example of a sample with a broad distribution of various compound classes, from short-chain organic acids (e.g. Lactic and Syringic acid) to long-chain alkanols (octacosanol) and a pentamethoxyflavone (a natural product found in many plants).

The terpenoids are dominated by compounds of the triterpene class, pentacyclic triterpenes with ursane or oleanane skeletons. These compound classes are found in ten of the specimen (Table 3 terpenoids column). They are widely distributed in nature primarily found in the cuticle waxes of many plants. Being rather non-specific they do however indicate waxy plant materials. Most of the short-chain organic acids detected, such as Gallic, Caffeic, Syringic, Malic, Citric and Quininic acid, are also of plant origin (cf. Fig. 6).

The steroids column in Table 3 contains compounds that in the non-targeted search showed fragmentation patterns characteristic of compounds containing a tetracyclic hydrocarbon skeleton, which has a steroid core structure. Sterols are not included here but under “Lipids” (Tables 3 and 4). Some of the components have been positively identified, e.g. Cholanoic and Allocholanoic acid (cf. Fig. 4), while others are only tentatively classified to possible molecular species, such as androstane, cholane, pregnane and lanostane derived compounds, based on characteristic fragments of trimethylsilyl derivatives^43^. This group of components deserves further exploration in future research.

**Figure 7:**
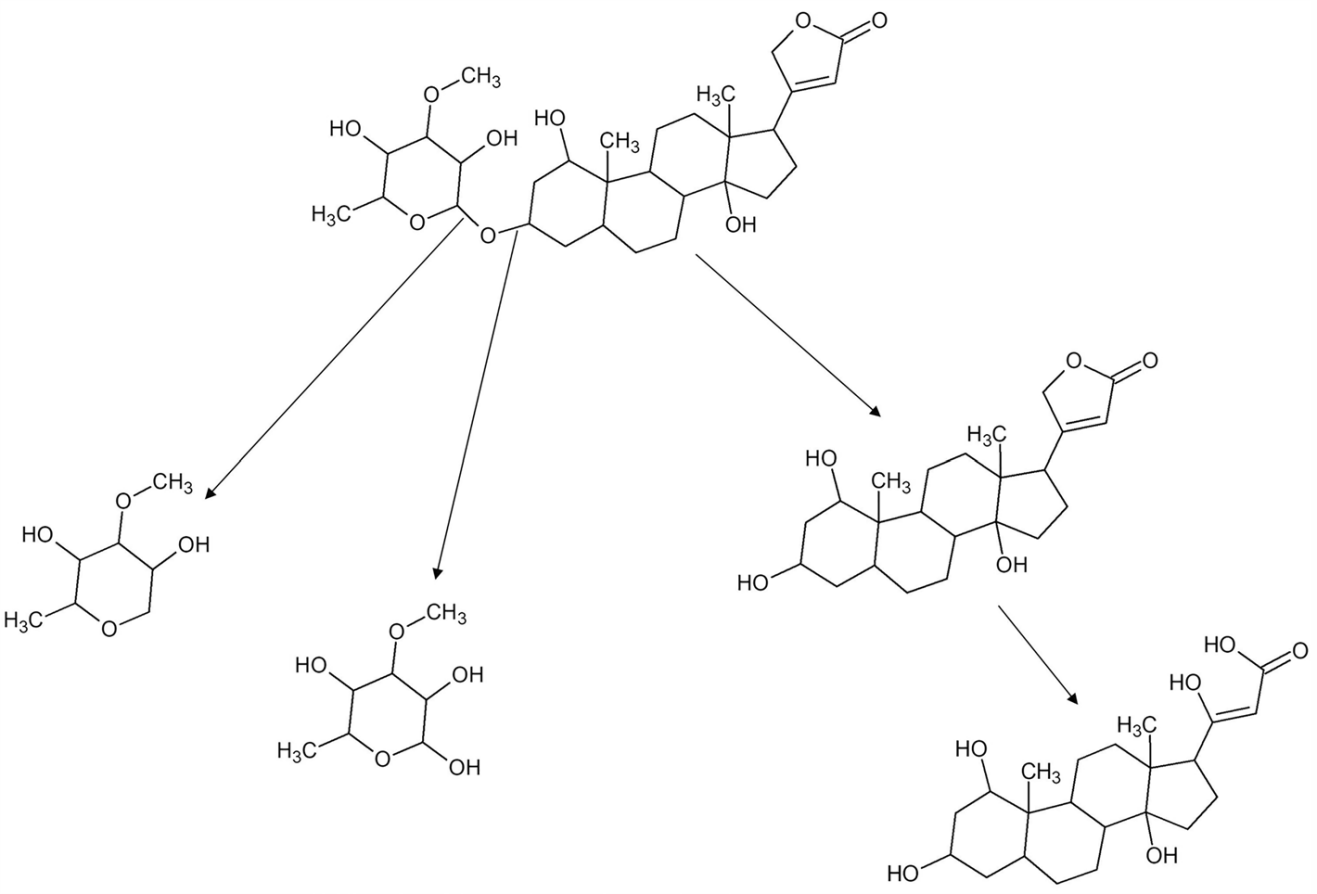
The hydrolysis of the cardiac glycoside Acovenoside A, leaving short-chain glucosidal residues and a steroid cardenolidal residue.

In most of the samples (n = 29) in this study there are plenty of mono- and disaccharide components that could derive from a hydrolytic process, such as would produce glucosidal residues. Cardiac glycosides are structurally based on a steroid core structure^44^. They are mostly C23-steroidal compounds, but there are variations, and they all have one of two ring structures connected to position 17 of the steroid core; a five-ring structure for cardenolides and a six-ring structure for bufadienolides. On the opposite end, at position 3 of the steroid core structure, is the glycosylation site where one or more sugar compounds are attached. These are not necessary for activity but serve to modify potency and duration of its effect. Cardiac glycosides used in known arrow poisons usually have only one sugar molecule, giving a rapid distribution to the heart and a short duration of activity. When such a molecule deteriorates the glycoside bond of the sugar compound could break due to hydrolysis, leaving short-chain glucosidal residues and a steroid cardenolidal residue (cf. Fig. 7).

These short-chain residues have a high solubility in water and would leach out of archaeological specimens during deposition in the soil. Also, the cardenolidal residues are polar due to the hydroxyl groups bound to the steroidal core. Steroidal compounds have been identified in the only archaeological sample of this study (BC11-13M, Fig. 4). The applicability of these biomarkers is environment dependent, so that the generally dry conditions of southern Africa may be conducive of preserving such biomarkers, different from wetter conditions in other regions. Although much work on these types of compounds are now conducted using Liquid Chromatography Mass Spectrometry, GC–MS is still the outstanding discovery tool in the area of steroids, especially in combination with MS/MS techniques such as Q-TOF (Quadropole-Time Of Flight MS)^43^. The localization of the hydroxyl groups on the steroidal core would be crucial for the identification of these residues and TMS derivatization and many of the fragment ions of this derivative give detailed information about hydroxyl group location providing good hopes for the positive identification of different molecular species.

## Discussion

A summary of the analytical results are presented in Table 5 wherein we highlight our interpretations of the FTIR and GC–MS outcomes as well as specimens with cardiotonic glycosides. Cardiac or cardiotonic glycosides are the active components of many plant species used in ethnographically documented arrow and dart poisons across the globe^14,22^. Triterpenoid and steroidal saponins as well as some alkaloids are cardio-active glycosides and can occur in all the parts of certain plants. The active components are mostly of the cardenolide type, which means that they are steroid derivatives to which a 5-membered unsaturated lactone ring is usually attached at the 17-position, but this may vary in some plants. Bisset^22^ also discusses how, besides glucose, many unusual sugars, not found elsewhere, are present, attached at the 3-position of the steroid skeleton. These may be methylated and may lack hydroxyl groups at the 2- and/or 6-positions. It is generally understood that the receptor for cardiac glycosides, the Digitalis receptor, is a membrane-bound enzyme that, amongst other things, acts as a pump to maintain the chemical balance of intracellular fluid^45^. When the cardiac glycosides bind to the enzyme, they interrupt the normal activity of the ‘pump’, and an overdose may result in arrhythmia and fibrillation. The *Apocynaceae* family represents the group of plants to which most of the cardiac-glycoside arrow-poison plants belong. The principal genera amongst these include *Acokanthera*, *Adenium*, *Beaumontia*, *Amaryllidaceae*, *Euphorbiaceae* and *Strophanthus*^22,46^. Apart from *Beaumontia*, taxa belonging to all these families are indigenous to southern Africa and are well-known ingredients in San hunter-gatherer poisons^10^. Ten of our specimens (Sample cluster 1 in Fig. 3) may have *Euphorbia* as a main ingredient according to the FTIR analysis.

**Table 5:** Summary of the hypothesized source materials for each of the arrow poisons.

	FTIR	GC–MS
BC11-13M	*Euphorbia*	Plant lipids and terpenes, steroidal compounds
ET1973/76/2	*Euphorbia*	Carbohydrates, plant oil
ET1989/15/9	Bees wax/*Diamphidia*	Plant terpenes
ET3420	*Adenium/Sansevieria*	Carbohydrates, plant oil
ET4706	*Euphorbia*	Carbohydrates, plant oil, animal fat
ET5099/3	*Adenium/Sansevieria*	Carbohydrates, plant oil
ET5107/5	*Euphorbia*	Carbohydrates, animal fat, steroidal compounds
ET63/33(f)	*Euphorbia*	Carbohydrates, plant oil and wax
ET6577	*Gum Arabicum*	Carbohydrates, plant oil, wax and terpenes
ET6632	*Gum Arabicum*	Carbohydrates, plant oil
ET88/117/4	*Euphorbia*	Carbohydrates, plant lipids
MA1942-256(J)	*Gum Arabicum*	Carbohydrates, plant lipids and terpenes, steroidal compounds
MA1948-61(I)	*Adenium/Sansevieria*	Carbohydrates, plant lipids
MA1965-3876	*Euphorbia*	Carbohydrates, plant lipids
MA4139 (K)	*Euphorbia*	Carbohydrates, plant oil
MM1-67-600	*Adenium/Sansevieria*	Plant oil, steroidal compounds
MM40-69-2235	*Adenium/Sansevieria*	Carbohydrates, plant oil, steroidal compounds
MM40-69-2607	Resin	Carbohydrates, plant wax, steroidal compounds
MM40-69-2805 (black)	Resin	Carbohydrates, plant oil and wax, animal fat, steroidal compounds
MM40-69-2805 (light)	*Gum Arabicum*	Carbohydrates, plant lipids and terpenes
MM40-69-2805 (transparent)	*Gum Arabicum*	Carbohydrates, plant terpenes, steroidal compounds, glue/lacquer
MM40-69-357	*Adenium/Sansevieria*	Carbohydrates, plant oil and terpenes, steroidal compounds
MM40-69-445	*Euphorbia*	Carbohydrates, plant oil
MM40-69-458	*Euphorbia*	Carbohydrates, plant lipids
MM649 G	*Adenium/Sansevieria*	Carbohydrates, plant oil, animal fat, steroidal compounds
MM649 K	*Adenium/Sansevieria*	Carbohydrates, animal fat, steroidal compounds
MM649 P	*Adenium/Sansevieria*	Carbohydrates, animal fat, steroidal compounds
SMP2528a	*Adenium/Sansevieria*	Carbohydrates, plant lipids and terpenes, steroidal compounds
SMP2528c	*Adenium/Sansevieria*	Carbohydrates, plant lipids and terpenes, steroidal compounds
SMP2528d	*Adenium/Sansevieria*	Carbohydrates, plant oil, animal fat
SMP loose	*Adenium/Sansevieria*	Carbohydrates, plant lipids and terpenes, steroidal compounds

“Steroidal compounds” designate samples with potential cardenolidal residues.

Thirteen specimens (Sample clusters 2 and 3 in Fig. 3) may have *Adenium* as a main ingredient. *Adenium* species contain highly toxic cardiotonic glycosides^22^. *Adenium miltiflorum* (impala lily) is widely known in Africa as a source of fish and arrow poison^11^, and *Adenium boehmianum* from northern Namibia is known to be the source of an extremely toxic arrow poison^14^. *Adenium* poisons are mostly prepared from the bark, but sometimes also the roots. In east Africa it is often an ingredient used in combination with other poisonous ingredients to form a compound poison^47^. For example, Neuwinger^14^ reports that additives might include *Euphorbia* latex, and/or the sap of *Spirostachys africana* or *Aloe* species. In southern Africa it is also prepared on its own by, for example, Hei//om, Herero and Nama hunters of Namibia^25^, and Nadler^23^ reported that the Ju|wasi mixed *Adenium* sap with *Diamphidia* entrails.

Many *Euphorbia* species are used throughout Africa in arrow poison recipes^10,14^. The three species most commonly reported to be used as hunting poisons in southern Africa are the *E. ingens* (*E.Mey ex Boiss*), *E. virosa* and *E. arborescens*^25^, of which *E. virosa* is considered to be the most virulent^11^. To this list may be added *E. Tirucalli* (Linné) and *E. coerulescens*, both of which contain potent diterpenoids^12,14,48,49^. The carcinogenic latex contains various serine proteases^50^, terpenoids, lectins, and several esters of diterpene alcohols^14,50,51^. Among some of the tribes of the Namib and Kalahari *Euphorbia* poison is used in its simplest form, when the white milky latex is sundried to thicken and then directly applied to arrows^21^. The latex is, however, often mixed with other ingredients including *Acokanthera*^11^, *Boophane*^21^, *Adenium* and *Spirostachys africana* exudates^52^, as well as *Diamphidia* entrails^53,54^. The Hei||om and Ju|wasi near Grootfontein in Namibia use a complex recipe wherein *Euphorbia* exudate is mixed with *Strychnos* and *Boophane* extracts as additives to snake venom and *Diamphidia* poison^55^. This mixture is boiled for 10 min in a hollow stone into which the poison maker frequently spits during the intervals while chanting. We use this example to demonstrate the many complicating factors that need to be considered when analysing ancient poisons, and to highlight that most protocols will not be able to test for all variables so that most results will reflect only a portion of what may have been used.

*Sansevieria* species have a global distribution, all taxa tested were found to be toxic to mice^14^, and subsequent studies confirmed the presence of triterpenes, flavonoids and cardiac glycosides^56^. The cellulose rich leaves are used for fibre production. Poison makers of Namibia are known to add the juices of a heated leaf of *Sansevieria aethiopica* to strengthen and prolong the lifespan *Diamphidia*-based poison recipes, sometimes including other plant species such as *Protasparagus exuvialis*, *Swartzia madagascariensis*^14,23,57^. Hunters in northern Kenya simply smear the leaf juice of *Sansevieria* on already-poisoned arrows to ‘refresh’ the poison if it is thought to be too dry^14^. The use of *Sansevieria* exudates in arrow poisons may therefore have multiple purposes such as increasing toxicity, functioning as a binder as well as a re-activator.

The challenge of identifying ancient poison ingredients, as with most ancient organic molecules, is that they are prone to break down into smaller constituent molecular chains. It is a matter of some work to reconstruct these smaller chains into the correct parent compound. Complicating matters is the fact that most poison recipes included many different ingredients and have several preparatory steps. Here, we presented the biomolecular results of our three-step analysis of twenty-eight poisoned arrowheads, spanning the last 100 years. In addition, one 1000-year-old archaeological example was included to test the efficiency of our method on much older specimens.

ATR-FTIR and chemometrics proved to be useful for screening and general characterisation. The applied extraction and derivatisation protocol produce data on major components present in the poison samples. The resulting model is primarily able to separate and distinguish between different arrow poison binders, although the extractives of both fractions consist of a multitude of components from both binders and active substances.

Our results show that even when specimens are grouped according to region there is noticeable diversity in ingredients that were utilised in the poison recipes. This confirms ethnographic and historical observations^10,17^. It is worth noting however, that the younger specimens in our sample tended to contain more complex mixtures of sugars, peptides and lipids, whereas the older specimens, including the archaeological one from Kruger Cave, were dominated by lipids and terpenoids. Although our sample is relatively small and temporally restricted to the late 19th to mid-twentieth centuries, this does suggest that arrow poison recipes changed through time if not a result of decomposition. Our results indicate that plant extracts dominate the poison material, although, as has been noted previously, long-chain animal proteins are notoriously difficult to detect with GC–MS, even in their oxidative state, making it difficult to confidently rule out their presence in our samples. Another important finding is that the oxidative by-products of cardiac glycosides preserve and are detectible in the form of short-chain cardenolide residues. The identification of these residues on the Kruger Cave specimen means that this residue can be used as a biomarker of cardiac glycosides on archaeological arrow tips thought to have been poisoned.

Much work remains to be done to recognise the biodegradative pathways of other organic compounds and the effects of preparation procedures on such pathways. To this end, we plan to expand our research by incorporating additional poisons from southern African arrows housed at Kew Gardens, UK, and Etnografiska museet in Stockholm, with the purpose to create a solid methodological approach for the analysis of older archaeological samples. We also plan to test whether single compound stable carbon isotope analysis can distinguish certain animal-derived poisons like *Diamphidia* fat from plant and animal-based sources.

## Methods

### Sample selection

The ethno-historical material was collected from Museum Africa in Johannesburg, Ditsong Culture History Museum in Pretoria, and the KwaZulu-Natal Museum in Pietermaritzburg (Table 6). Common to most of these collections is the lack of detailed provenance information. The arrows were mostly collected and/or donated to the museums by private collectors in the early twentieth century. The material from Ditsong and Museum Africa come from northern and central Namibia and from the northern Kalahari, which encompasses the eastern portion of Namibia and the western half of Botswana (Fig. 1). In most cases the particular groups from whom the arrows were collected are not mentioned—with ‘San’ or ‘Bushman’ being the broad designation—but in all probability were made by the Ju/wasi. However, in the case of the Fourie collection (designated by the prefix MM in Table 6), we know the arrows were collected from the Hai//om in northern Namibia, and, unlike the other collections, we have plenty of provenance information for these arrows^52,58,59^.

**Table 6:** Arrowheads sampled for poison residues.

Accession #	Attribution	Region	Age	Description
BC 11-13M	Later Stone Age	Magaliesberg	1020 ± 70 BP	Wood point
ET 1973/76/2	–	–	Pre 1955	Tanged metal arrow
ET 1989/15/9	!Kung	Northern Kalahari	Pre 1988	Poison applicator
ET 3420	–	Namibia	1920s	Tanged metal arrow
ET 4706	–	Namibia	Unknown	Tanged metal arrow
ET 5099/3	–	Namibia	1920s	Tanged metal arrow
ET 5107/5	–	Namibia	1920s	Bone point arrow
ET 63/33 (f)	San	Namibia	Pre 1963	Bone point arrow
ET 6577	Bushman	Kalahari	1923	Tanged metal arrow
ET 6632	San	Namibia	1923	Tanged metal arrow
ET 88/117/4	–	–	Pre 1988	Tanged metal arrow
MA 1942-256 (J)	San	Namibia	Early twentieth century	Tanged metal arrow
MA 1948-61 (I)	San	Namibia	Early twentieth century	Tanged metal arrow
MA 1965-3876	San	Namibia	Early twentieth century	Tanged metal arrow
MA 4139 (K)	Hai//om	Northern Namibia	Pre 1936	Tanged metal arrow
MM 1-67-600	San	Namibia	Pre 1967	Bone point arrow
MM 40-69-2235	Hai//om	Northern Namibia	1920s	Tanged metal arrow
MM 40-69-2607	Hai//om	Northern Namibia	1920s	Poison applicator
MM 40-69-2805	Hai//om	Northern Namibia	1920s	Poison applicator
MM 40-69-357	Hai//om	Northern Namibia	1920s	Bone point arrow
MM 40-69-445	Hai//om	Northern Namibia	1920s	Tanged metal arrow
MM 40-69-458	Hai//om	Northern Namibia	1920s	Tanged metal arrow
MM 649 G	Hai//om	Northern Namibia	1920s	Bone point arrow
MM 649 K	Hai//om	Northern Namibia	1920s	Bone point arrow
MM 649 P	Hai//om	Northern Namibia	1920s	Bone point arrow
SMP 2528a	Bushman	Drakensberg	19th–20th century	Bone point metal inset
SMP 2528c	Bushman	Drakensberg	19th–20th century	Bone point metal inset
SMP 2528d	Bushman	Drakensberg	19th–20th century	Bone point metal inset
SMP loose	Bushman	Drakensberg	19th–20th century	Loose material

Information provided in the table comes from the museum accession catalogues. SMP loose is the accumulated loose material from the Vinnicombe hunting kit that had flaked off the arrows while in storage.

The arrows from the KwaZulu-Natal Museum come from the so-called Vinnicombe Collection. These arrows were found in a leather quiver together with a complete hunting kit in Eland Cave in the Drakensberg Mountains by Johannes Lombard in 1926^60^. The collection of poisoned arrows is one of only two that have been found in the Drakensberg region. Finally, we include a poisoned wood arrowhead recovered from a stratified context, carbon dated to 1020 ± 70 BP, at Kruger Cave^61^. Kruger Cave is located in the Magaliesberg, South Africa (Fig. 1), a region for which no ethno-historical information on hunting poisons exists. This arrowhead, and two others from the site, are among the few obviously poisoned arrows recovered from archaeological contexts^13^.

We sampled poison from four varieties of arrows (Fig. 1)^62,63^. Arrows were selected which had bits of poison flaking off and which were therefore easy to sample without causing much damage. Approximately 1 mg of material was removed from each arrowhead under sterile conditions.

### Attenuated total reflectance Fourier transform infrared spectroscopy

The samples of arrow poison residues, and reference samples were analyzed by Attenuated Total Reflectance Fourier Transform Infrared Spectroscopy (ATR FTIR). This technique was chosen since it allows the analysis of very small samples (< 0.1 mg). The choice of reference materials for the model was selected based on known arrow poison ingredients and on the result of comparing sample spectra with reference materials in our spectral database. The selection of known ingredients is primarily based on southern African sources^10^ but also include materials known from other regions^14^. The final model was based on 52 reference materials consisting of various plant tissue (n = 24, including *Adenium*, *Euphorbia* and *Sansevieria*), plant resins (n = 6), gum arabic (n = 6), pupae of the poison arrow beetle (*Diamphidia*, n = 6), bees wax (n = 4) and different animal glues (n = 6). The spectral data obtained from the ATR FTIR analyses of the samples were first investigated for groupings using a hierarchical cluster analysis^31^. Then the data was further processed using a combination of PCA and DFA. The PCA is here used as a data reducing technique. The spectral loading of the first principal components (PCs) are investigated to determine their diagnostic relevance. Relevant PCs are then used to build a DFA model based on the PCs from the reference materials. The accuracy of the model is tested and then used to classify the samples. This processing was performed using the Statistica 12 software package.

The ATR FTIR instrument used in this study was a Thermo Scientific Nicolet iS10 FTIR equipped with a diamond crystal ATR accessory. The IR-spectra were recorded between 4000 and 525 cm^−1^, using 32 scans with a resolution of 4.0 cm^−1^. The resulting IR-spectra were exported as csv-files for arithmetical analysis.

### Gas chromatography–mass spectrometry

Solvent extractable components were then analyzed by GC–MS, using an ultrasonic aided solvent-extraction. Samples of the arrow poison residues of a few milligrams were homogenized through sonication in a few hundreds of microliters of a mixture of chloroform and methanol (2:1, v:v). The non-extractable residue and the liquid phase were separated through centrifugation (3000 rpm, 30 min) and the liquid phase was collected. This process was repeated three times and the extracts were combined.

The use of methanol as a polar component in the extraction mixture is important for the efficiency of this method as it improves the solubilization of lipid molecules^64^ but it also facilitates the extraction of many non-lipid polar compounds. As a result, the extracts may contain a large range of organic compound classes, both apolar (e.g., lipids, terpenes, etc.) and polar (e.g., sugars, dicarboxylic acids, etc.). The solvent was removed by a gentle stream of nitrogen gas and the dried extracts were treated using *N*, *O*-bis(trimethylsilan)trifluoroacetamide (BSTFA) with 10% chlorotrimethylsilane at 70 °C for 20 min. This procedure blocks protic sites on polar and apolar compounds alike, improving their properties for GC–MS analysis^43^. Access reagent was removed using a gentle stream of nitrogen gas and the silylated components were re-dissolved in 100 µl of n-hexane and analyzed by GC–MS.

The silylated components from the solvent wash were analyzed using a HP 6890 Gas Chromatograph equipped with a SGE BPX5 capillary column (30 m × 220 µm × 0.25 µm). The injection was done by pulsed splitless (pulse pressure 25 Psi) technique at 325 °C using an Agilent 7683B Autoinjector. The injection volume used was 1.0 µl. The oven was temperature programmed with an initial isothermal of 2 min at 50 °C, followed by an increase of the temperature with 10 °C per minute to 360 °C, followed by a final isothermal at this temperature for 15 min. Helium was used as a carrier gas and held at a constant flow of 2.0 ml per minute throughout the analysis. The gas chromatograph was connected to a HP 5973 Mass Selective Detector via an interface with a constant temperature of 360 °C. The fragmentation of separated compounds was done by electronic ionisation (EI) at 70 eV. The temperature at the ion-source was 230 °C. The mass filter was set to scan between m/z 50 and 700, providing 2.29 scans per second. The temperature of the mass filter was 150 °C. The results were evaluated using the MSD Chemstation software, as well as the Masshunter 10 software together with the NIST Mass Spectral Search Program 2.3 and the NIST 2017 library. Investigating the samples with a non-targeted approach meant using the Masshunter software to identify peaks in the chromatograms and extract background subtracted mass spectra from these peaks. These mass spectra were then investigated using the NIST Mass Spectral Search Program aiming at identifying the compounds giving rise to the peak(s) and mass spectra, together with a comprehensive review of the mass spectral fragmentation of trimethylsilyl derivatives^43^.

## Data Availability

The datasets generated and analyzed for this study can be found in the Swedish National Data Service: Data of potential biomarkers for southern African hunter-gatherer arrow poisons applied to ethno-historical and archaeological samples | Swedish National Data Service (gu.se).
